# An Alternative Perspective for the Theory of Biological Control

**DOI:** 10.3390/insects9040131

**Published:** 2018-10-02

**Authors:** Nicholas J. Mills

**Affiliations:** Department of Environmental Science, Policy and Management, University of California, Berkeley, CA 94720-3114, USA; nmills@berkeley.edu; Tel.: +1-510-642-1711

**Keywords:** pest, weed, importation, establishment, impact, demography, genetics

## Abstract

Importation biological control represents the planned introduction of a specialist natural enemy from the region of origin of an invasive pest or weed. For this study, the author considered why attempts to develop a predictive theory for biological control have been misguided and what future directions might be more promising and effective. Despite considerable interest in the theory of consumer–resource population dynamics, such theory has contributed little to improvements in the success of biological control due to a focus on persistence and equilibrium dynamics rather than establishment and impact. A broader consideration of invasion biology in addition to population ecology offers new opportunities for a more inclusive theory of biological control that incorporates the demographic and genetic processes that more specifically address the establishment and impact of introduced natural enemies. The importance of propagule size and genetic variance for successful establishment, and of contributions to host population growth, relative population growth rates, interaction strength, and coevolution for suppression of host abundance are discussed as promising future directions for a theory of biological control.

## 1. Introduction

Biological control is an ecosystem service in which a pest or weed is effectively controlled through interactions with its natural enemies, and in many cases the natural enemies are insects [1]. The control of alien invasive species through the deliberate introduction of specialist natural enemies from their geographic region of origin, known as importation biological control, serves as one of the clearest examples of the value of biological control as an ecosystem service [2,3]. Classic cases include successful control of the cottony cushion scale (*Icerya purchasi* Mask.) in California following the introduction of the vedalia beetle (*Rodolia cardinalis* Muls.) from Australia [4], of the cassava mealybug (*Phenacoccus manihoti* Matile-Ferrero) in Africa following the introduction of a parasitoid wasp (*Anagyrus lopezi* De Santis) from South America [5], and of St. John’s wort (*Hypericum perforatum* L.) in New Zealand following the introduction of the leaf beetle (*Chrysolina hyperici* [Först.]) from Europe [6]. Nonetheless, failure to achieve at least some level of control has continued to be a frequent outcome for importation biological control, for example, 74% (1764 of 2384) failure for natural enemy species used against arthropod pests [2] and 62% (277 of 449) failure for those used against weeds [3].

Importation biological control, hereafter referred to simply as biological control, began as a very pragmatic and empirical approach to pest and weed management in the late 1800s, and while we have made great strides in terms of our knowledge of the ecological basis for this practice since then [1], there is little evidence for a significant improvement in rates of success over time [2,3]. Perhaps the most disappointing aspect of this historical record is that although a theoretical explanation for success versus failure has been “a relentlessly pursued but elusive goal” [7], it has achieved “few if any, general principles, or even rules of thumb, to guide the efforts of biological control” [8]. McEvoy [9] makes a compelling case for the potential of ecological theory to improve the effectiveness and safety of biological control, and for this study, the author considered why attempts to develop a predictive theory for biological control have been misguided and what future directions might be more promising and effective.

## 2. Population Dynamics and the Paradox of Biological Control

The development of consumer–resource theory in ecology has a long history with a notable bias toward parasitoid–host models as the simplest example of a tightly coupled consumer–resource interaction [8,10]. When a host is successfully attacked by a female parasitoid, the host dies and is converted into either a single (solitary parasitoids) or a mean number (gregarious parasitoids) of offspring in the next generation, which greatly simplifies the coupling of host and parasitoid populations in simple mathematical models. In contrast, the effects of predation and herbivory on the dynamics of consumer and resource populations are more complex, as there is no simple linkage for these interactions between resource and consumer fitness. Consequently, predator–prey and herbivore–plant models have attracted rather less attention in the context of a theory for biological control [11,12,13]. For example, most of the models developed for weed biological control are stage-structured matrix models of the weed population alone with insect herbivory included as a reduction in per capita seed production [14], and only a few models directly couple the interaction of both herbivore and weed populations [15].

Successful biological control of pests and weeds has two important characteristics: (1) the persistence of both natural enemy and host populations and (2) a sustained reduction in the size of the host population. The simplicity of coupled parasitoid–host models has been a particularly attractive framework for addressing these two characteristics of biological control because analytical solutions can often be found for equilibrium abundance of host and parasitoid populations and for the local stability of these equilibria. The Nicholson–Bailey model has played a pivotal role in the theory of parasitoid–host population dynamics [10], but the inherent instability of this discrete-time model (representing insects with a single generation each year) inevitably leads to the extinction of one or both populations. This lack of persistence drove theoreticians to focus almost exclusively on a quest to find life history traits and population structures that could account for local stability and consequently the persistence of biological control interactions [16]. Although continuous time models with neutral stability were also developed (representing insects with multiple overlapping generations each year) [8], the quest to explain persistence remained strong and dominated the theory of parasitoid–host interactions. Implicit in this theory are two key assumptions. The first is that parasitism must introduce top-down density dependence to provide local stability for the interaction, a notion that has also been applied to herbivore–plant interactions with a suggestion that herbivores may need to alter the strength or form of density dependence in plant populations to achieve successful biological control [17]. The second is that parasitoid–host interactions reach equilibrium conditions under natural field conditions, a simplification that facilitates the analysis of the local stability properties of the equilibria and comparison of host equilibrium densities among nested models.

### 2.1. Local versus Regional Persistence

**Figure 1:**
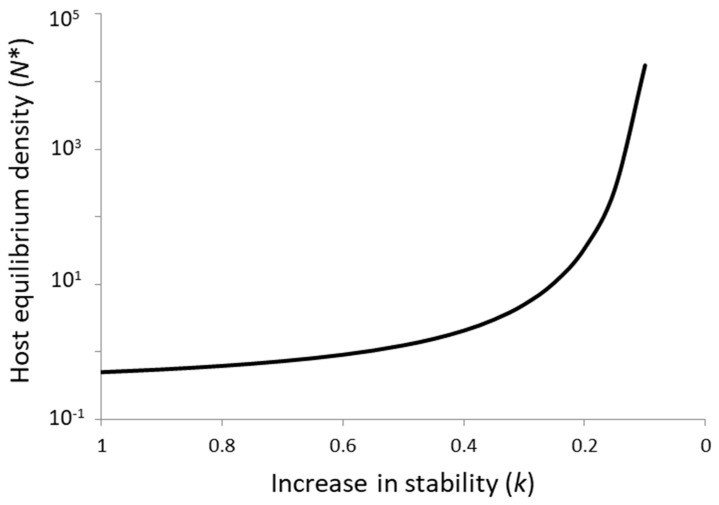
The paradox of biological control as illustrated by the Getz–Mills model [20], a discrete-time parasitoid–host model with aggregated (and host-density-independent) encounters between parasitoids and hosts and an attack strategy that varies from search limitation to egg limitation. The paradox is represented by the tradeoff between increased stability (greater aggregation of encounters, k→0) and host equilibrium density (N*).

With regard to the first key assumption of parasitoid–host theory, it is well known that persistence can also occur at a regional scale in the absence of local stability due to asynchronous dynamics among subpopulations in a spatially fragmented environment [18]. Persistence can be increased either by limited dispersal between a large set of subpopulations, by random dispersal decoupled from subpopulation densities, or by suboptimal parasitoid foraging strategies (host-density-independent aggregation). Irrespective of whether the persistence of parasitoid–host interactions under field conditions is driven at a local or regional scale, however, theory presents a clear paradox for biological control in that almost all models exhibit a tradeoff between persistence and host suppression [8,19]. This is well illustrated by the Getz–Mills model [20] (Figure 1), a discrete-time model for a host with no self-limitation and a parasitoid that varies between search and egg limitation combined with an aggregated distribution of host encounters that is host-density-independent. The more aggregated the parasitoid encounters with hosts (k→0), the stronger the stabilizing effect and the less effective the parasitoid is in the suppression of host density. Or conversely, the more effective the parasitoid is in suppressing host equilibrium density below the carrying capacity set by resource availability, the more likely it is that the system will be unstable.

Unfortunately, there has been very little support for follow-up studies, under field conditions, of the difference between success and failure in biological control, and consequently there have been few applications that provide sufficient data to be able to address the question of local stability versus regional persistence. For two of the most detailed studies of biological control success, the introduction of a parasitoid (*Aphytis melinus* DeBach) for control of California red scale (*Aonidiella aurantii* Mask.) does provide evidence for local stability [21], but the introduction of the cinnabar moth (*Tyria jacobaeae* (L.)) and the ragwort flea beetle (*Longitarsus jacobaeae* (Waterhouse)) for control of tansy ragwort (*Jacobaea vulgaris* (Gaertn.)) provides evidence of instability at a local scale [9]. In addition, Murdoch et al. [22] found no evidence for local stability in the parasitoid–host interactions associated with successful biological control of winter moth, olive scale, larch sawfly, and walnut aphid. It is also interesting to note that Kean and Barlow [23] found that a spatially explicit metapopulation model allowed for a greater level of host suppression at equilibrium than a corresponding non-spatial model, suggesting that regional persistence may be more compatible with host suppression than local stability. Consequently, if success in biological control is more generally represented by metapopulation dynamics than by local dynamics, then a shift in the focus of biological control theory from local population processes to natural enemy foraging behavior and the response of individuals to the patchy distribution of hosts in a spatially fragmented environment may be needed [24].

### 2.2. Transient versus Equilibrium Dynamics

With regard to the second key assumption of parasitoid–host theory, it is questionable whether equilibrium dynamics ever exist in natural communities of interacting species due to both seasonal and annual changes in environmental conditions. This may be further exacerbated for biological control in agricultural systems due to frequent management disturbance. Under these circumstances, shorter-term transient dynamics may better represent biological control than longer-term equilibrium dynamics [25,26], and Kidd and Amarasekare [26] demonstrate that the parasitoid traits which promote host suppression differ under transient and equilibrium conditions. For example, greater suppression of transient host abundance results from a shorter handling time (or higher fecundity) which allows saturation of the functional response at higher host densities. In contrast, greater suppression of equilibrium host abundance results from a greater conversion efficiency of hosts attacked to parasitoid progeny produced. Thus, the extent to which an equilibrium theory of parasitoid–host population dynamics has general applicability to the practice of biological control is open to question.

The strong focus on persistence and equilibrium dynamics in models of parasitoid–host interactions has led to a mismatch between theory and observation for biological control. Theory predicts that the more successful a natural enemy is in suppressing the abundance of its host below the carrying capacity of the environment, the less persistent it will be. In contrast, from the historical record of biological control we can find many examples of persistent suppression of pest and weed abundance by introduced natural enemies under field conditions. The consequence of this mismatch is that there are few general principles, or rules of thumb, to guide the application of biological control to greater levels of success. This is unfortunate, as it has delayed further progress in the development of a theory for biological control success. Therefore, there may now be much to be gained from an alternative perspective that is more inclusive of theoretical developments in other relevant fields of study.

## 3. Invasion Biology and the Theory of Biological Control

**Figure 2:**
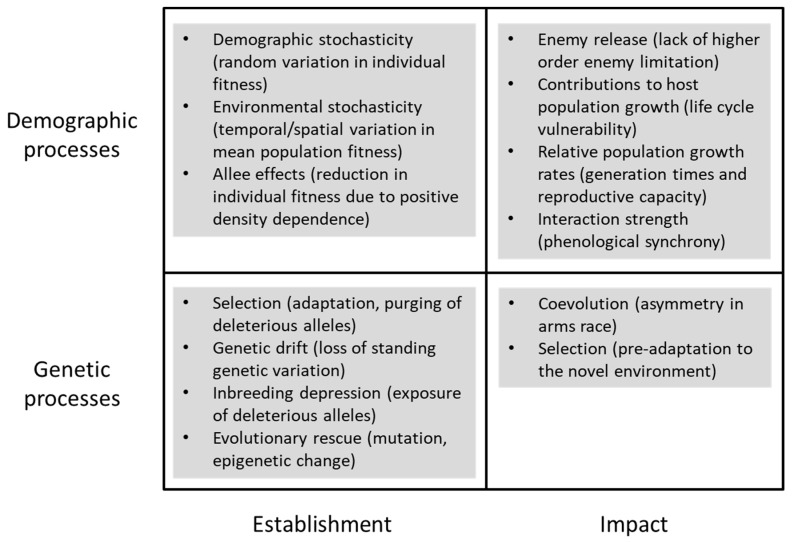
Demographic and genetic processes that are of importance to the development of a more inclusive theory of biological control.

In addition to its relevance for population ecology, biological control is an example of a planned invasion of an exotic natural enemy for control of an invasive pest or weed [1,27]. Recent advances in invasion biology provide theoretical developments that are directly applicable to biological control and should be considered in the development of a more inclusive theory for biological control (Figure 2). There are also several advantages that the integration of invasion biology and population ecology can bring to the application of biological control. For example, invasion biology provides a unified framework for the invasion process that not only includes biological control as a special case, but also specifically addresses the barriers to successful establishment of an exotic species in a novel environment [28]. In addition, the realization that evolutionary dynamics can occur over ecological time scales, as exemplified by invasion events [29], has encouraged consideration of genetic as well as demographic processes in invasion biology [30]. In contrast, population ecology has continued to focus on how and why population densities change in time and space, demographic processes such as regulation, and the dynamics of species interactions [31]. Population ecology is thus central to understanding the impact of introduced natural enemies in the context of biological control, but has contributed rather less to understanding establishment. Thus, the integration of theoretical developments from both invasion biology and population ecology could better illuminate and inform the practice of biological control.

### 3.1. Dynamics of Natural Enemy Establishment

Invasive pests and weeds that become targets for biological control represent an abundant resource for introduced natural enemies, but the success of natural enemy establishment in a novel environment is influenced by both demographic and genetic processes that affect small founder populations [30,32] (Figure 2). The most important demographic processes for founder populations are demographic stochasticity, environmental stochasticity, and Allee effects. In addition, key genetic processes such as drift and inbreeding depression can reduce the standing genetic variation among individuals and consequently the mean fitness of a founder population in the absence of adaptation. For both demographic and genetic processes, theory predicts that the probability of establishment should increase with the size of the founder population by buffering stochastic effects and genetic variation, and by reducing Allee effects and inbreeding [30,33,34,35]. There is strong empirical evidence from the invasion biology literature that propagule pressure, namely, the total number of individuals introduced to an area, is the most consistent predictor of establishment success [36]. Propagule pressure has also been shown to influence the establishment of natural enemies used for biological control introductions [37,38]. For example, an analysis of 515 releases of 254 parasitoid species used for biological control introductions showed a significant positive influence of propagule pressure on establishment success [37]. In a more detailed study, Duncan et al. [38] analyzed the success of establishment of a psyllid (*Arytainilla spartiophila* (Först.)) for the control of broom (*Cytisus scoparius* (L.)) in New Zealand from a field experiment in which propagule pressure was manipulated across a series of different release sites [39]. They found that the relationship between probability of establishment and propagule pressure was well described by a model that included the effects of demographic stochasticity and the net balance between the contributions of an Allee effect and heterogeneity in environmental conditions. A stronger contribution from environmental heterogeneity obscured any influence of an Allee effect and resulted in a disproportionate failure of establishments from psyllid releases of larger rather than smaller propagule pressure.

It is also important to recognize that propagule pressure consists of two interacting components: propagule size, or the mean number of individuals per introduction event, and propagule number, or the number of introduction events. Whereas some theoretical studies suggest that propagule size is more important for establishment success, even in the presence of environmental variability [40], others suggest that propagule number may be more important than size [41,42]. It can be argued that the probability of at least one introduction event leading to establishment is a function of the average per-release establishment rate and propagule number [41]. Consequently, for a natural enemy species that readily establishes in novel environments, propagule size could be more important than propagule number, whereas for a natural enemy species with a poor record of establishment, the reverse would apply. In analyzing establishment records for 74 weed biological control agents in the state of Oregon, USA, there was no evidence for an effect of propagule size, but good evidence that propagule number was important in achieving a high probability of establishment, suggesting that it may often be suboptimal in weed biological control programs [43].

The influence of genetic processes on the success of establishment has not been studied as extensively as the influence of demographic processes. However, theory has suggested that inbreeding depression is likely to be less important than demographic processes in influencing establishment [40], and there is some supporting evidence from an experimental laboratory study with red flour beetles [44]. In contrast, however, another recent experimental study with red flour beetles suggests that either preadaptation to a novel environment, or increased genetic variation that includes at least some individuals that are preadapted, can be more important than propagule size in increasing the probability of establishment of founder populations [45].

These recent developments provide valuable guidelines for enhancing natural enemy establishment by increasing propagule number and increasing the genetic variation among individuals used for biological control introductions. The extent of preadaptation to climatic conditions can also be enhanced through the use of species distribution models to assess the climatic match of source and target regions for natural enemy introductions [46] or to select locations where surveys for natural enemies should be conducted [47]. One question that has yet to be resolved in this context is whether founder populations for introduction should be selected from sources with the closest genetic match (similarity of host genetic backgrounds for the natural enemy) or the best climatic match (similarity of host environments for the natural enemy) or under which conditions one or the other may lead to a more successful establishment.

### 3.2. Dynamics of Natural Enemy Impact

Whether or not an established natural enemy has sufficient impact to suppress the abundance of a target host seems to be determined primarily by characteristics of the interaction of natural enemy and host populations. Setting persistence aside, we can then ask what factors are most likely to drive the impact of introduced natural enemies (Figure 2). It is very clear, at least for plant populations, that some of the best examples of strong impact arise from accidental introductions of exotic insect pests [48] and result either from enemy release and/or the absence of coevolved plant defenses. In addition, the current failure of a previously successful program to control Argentine stem weevil (*Listronotus bonariensis* (Kushel)) in New Zealand with an introduced parasitoid (*Microctonus hyperodae* Loan) also highlights the importance of coevolution [49]. In this case, the sexually reproducing weevil has been able to respond to the strong selection pressure imposed by the asexually reproducing parasitoid and to evolve resistance to parasitism over a period of as little as seven years. This clearly raises the question of whether the lower level of standing genetic variation associated with asexual versus sexual reproduction can compromise the coevolutionary arms race between virulence and resistance in biological control. It may also account for why asexual reproduction in weeds has been identified as one of the most important characteristics for success of weed biological control [50].

Theoretical studies of post-establishment processes in invasion biology have focused heavily on rates of spread and, in the context of biological control, on the potential for an established natural enemy to reduce, stop, or even reverse the spread of an invasive host [51]. Although this is a valuable aspect of biological control, it makes the assumption that an established natural enemy does have sufficient impact on the abundance of the target host, at least at a local scale. To address the dynamics of natural enemy impact more broadly in the context of the success and failure of biological control, however, requires additional insights from population ecology. As discussed earlier, theoretical studies of consumer–resource dynamics have been addressed extensively, but have provided few guidelines for biological control. As a consequence, the author focuses here on three less well-known demographic effects as potential drivers of success that deserve closer examination in the future: contributions to host population growth, relative population growth rates, and interaction strength. The significance of natural enemy impact as a contribution to the suppression of host population growth can often be buffered by compensatory processes, such as weed regrowth and/or density dependence [52]. Consequently, the notion of vulnerability in the life cycles of target weeds and pests has become of increasing interest [9,53,54,55] to ensure that the damage imposed by a selected insect herbivore or the mortality caused by a selected parasitoid translates into the greatest reduction in population growth of the target host. So far, such models have not incorporated the effects of density dependence [17,56], but they represent an important step forward in addressing how best to maximize the impact of introduced natural enemies on the suppression of host population growth. While it is too early to know how valuable this approach might be in practice, it has been very informative in identifying the importance of the simultaneous effects of herbivory from the ragwort flea beetle and the interspecific competition from background vegetation in suppressing incipient outbreaks of tansy ragwort [9,55].

Another way in which the impact of a natural enemy can be maximized is through its relative population growth rate. To have a substantial impact, a natural enemy must have some form of numerical advantage over the host, such as a shorter generation time [8,57,58] or a greater reproductive capacity [25], to allow its population to capitalize on host availability and respond rapidly to changes in the host population growth rate. Despite the intuitive appeal of this simple notion, relative population growth rate has only rarely been considered as a potential driver of biological control success and deserves greater attention because it also provides a very simple criterion for the comparison and selection of candidate natural enemies for introduction.

**Figure 3:**
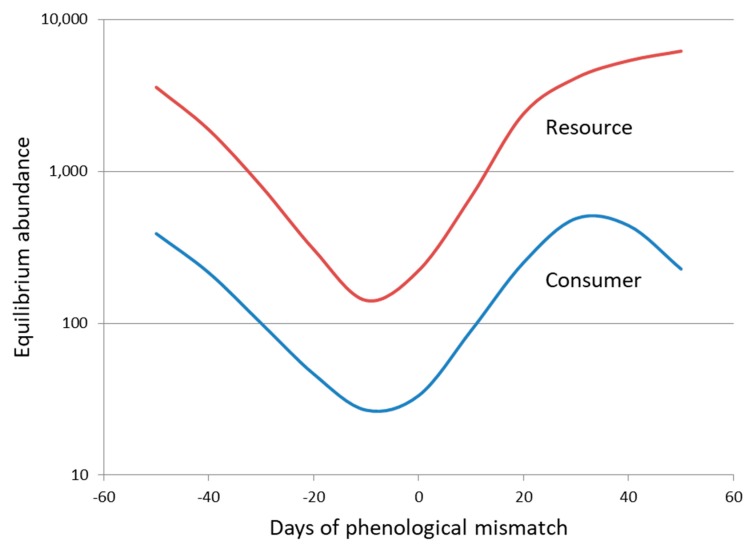
The influence of phenological mismatch on the equilibrium abundance of consumer and resource populations for a model in which within-year dynamics is continuous, between-year dynamics is discrete, and phenological mismatch is represented by a difference in the mean dates of normal distributions for recruitment of consumer and resource populations. Redrawn after [68].

A third aspect of natural enemy–host interactions that has been neglected as a key influence on the suppression of host population abundance is that of interaction strength and how it is affected by temporal synchrony. Discrete-time models assume that natural enemy and host populations are perfectly synchronized, and although continuous-time models often include stage structure and stage durations [8,16], the analysis of these models has remained closely focused on persistence rather than interaction strength. In contrast to theory, many of the natural enemies that are used for biological control introductions utilize relatively short-lived stages in the life cycles of the insect pests or weeds that they attack and so consequently are vulnerable to mismatches in phenology as they respond to abiotic cues in novel environments [59,60,61]. Phenological synchrony has been of increasing interest in recent years due to a need to understand the link between climate change and the demography of trophic interactions [61,62,63,64]. Two interesting aspects of this work are that the tendency for phenology to shift earlier in the year in response to climate warming declines with trophic level from plants to predators [63], and that mismatches in phenology have been better documented for insect herbivores [65] than for insect parasitoids [66]. From a theoretical perspective, phenological asynchrony creates a partial temporal refuge from natural enemy attack and acts as a source of stability in parasitoid–host models, such that greater asynchrony leads to increased equilibrium density of the host population [67]. A more recent theoretical model suggests that a similar or slightly earlier phenology for the consumer than the resource population (negative mismatch) leads to the greatest level of resource exploitation and consequently the lowest equilibrium abundance for both populations [68] (Figure 3). In contrast, too early a phenology for the consumer or a phenology that is later than that of the resource population leads in both cases to larger consumer and resource populations. Thus, an avoidance of mismatch in the temporal synchronization of introduced natural enemies with their target hosts in novel environments may prove to be one of the most important challenges for biological control. Interaction strength may well drive the difference between success and failure in the suppression of host abundance and be influenced by the breadth of the temporal window of host vulnerability, its temporal match to natural enemy flight periods, and the variability in phenology among individuals of both populations.

## 4. Conclusions

Through the history of biological control, theory has been rather narrowly focused on the demography of parasitoid–host interactions at equilibrium. In addition, undue attention to persistence rather than impact in the dynamics of parasitoid–host interactions has limited the contribution of theory to the improvement of success rates in biological control. A more inclusive theory for biological control needs to embrace the importance of genetic as well as demographic processes as influences on both the establishment of founder natural enemy populations and the suppression of host populations in novel environments. Biological control can no longer afford to remain a largely pragmatic approach to pest and weed management that is uninformed by a predictive and testable theory. For too long, we have relied on the outcomes of individual case studies to guide the application of biological control. To make further progress, there is a clear need not only to be informed by the insightful deductions that can be made from realistic population and evolutionary models, but also to adopt an experimental as well as observational approach to the application of biological control [9,69]. A stronger linkage between theory and application will be needed for biological control to develop as a more predictive science, and the integration of concepts from both invasion biology and population ecology offers an alternative perspective that incorporates a broader range of theory and experimental methodology. Greater emphasis on the importance of genetic as well as demographic processes as central components of biological control is needed, and if more widely adopted, this could lead to substantial improvements in the success and safety of future applications.
